# On the comparison of regulatory sequences with multiple resolution Entropic Profiles

**DOI:** 10.1186/s12859-016-0980-2

**Published:** 2016-03-18

**Authors:** Matteo Comin, Morris Antonello

**Affiliations:** Department of Information Engineering, University of Padova, Padova, Italy

**Keywords:** Alignment-free, Sequence comparison, Entropic profiles

## Abstract

**Background:**

Enhancers are stretches of DNA (100–1000 bp) that play a major role in development gene expression, evolution and disease. It has been recently shown that in high-level eukaryotes enhancers rarely work alone, instead they collaborate by forming clusters of *cis*-regulatory modules (CRMs). Although the binding of transcription factors is sequence-specific, the identification of functionally similar enhancers is very difficult and it cannot be carried out with traditional alignment-based techniques.

**Results:**

The use of fast similarity measures, like alignment-free measures, to detect related regulatory sequences is crucial to understand functional correlation between two enhancers. In this paper we study the use of alignment-free measures for the classification of CRMs. However, alignment-free measures are generally tied to a fixed resolution *k*. Here we propose an alignment-free statistic, called $EP^{*}_{2}$, that is based on multiple resolution patterns derived from the Entropic Profiles (EPs). The Entropic Profile is a function of the genomic location that captures the importance of that region with respect to the whole genome. As a byproduct we provide a formula to compute the exact variance of variable length word counts, a result that can be of general interest also in other applications.

**Conclusions:**

We evaluate several alignment-free statistics on simulated data and real mouse ChIP-seq sequences. The new statistic, $EP^{*}_{2}$, is highly successful in discriminating functionally related enhancers and, in almost all experiments, it outperforms fixed-resolution methods. We implemented the new alignment-free measures, as well as traditional ones, in a software called *EP*-*sim* that is freely available: http://www.dei.unipd.it/~ciompin/main/EP-sim.html.

## Background

How to measure the degree of similarity between biological sequences is one of the foremost questions on the mind of bioinformaticians. This problem relates to the identification of homologous sequences like proteins and, to this end, the use of tools like BLAST is nowadays a standard procedure. In this paper we study the same question but for regulatory sequences such as promoters or enhancers of genes. The detection of similarities between coding sequences is a widespread approach to estimate functional correlations. Indeed, there is a general belief that similar binding site contents in regulatory sequences are expected to drive similar expression patterns [1]. Moreover, large collections of regulatory sequences have become available after the advent of ChIP-seq technologies and the identification of sequences regulating the same cell-type in the analysis of ChIP-seq data is definitely a crucial step.

Many articles [1] discuss recent views on enhancers or cis-regulatory modules (CRMs), one of several types of genomic regulatory elements, and their coordinated action in regulatory networks. Enhancers are stretches of DNA (100–1000 bp) that play a major role in the development of gene expression. They can upregulate, i.e. enhance, the transcription process driving animal development. A single cell can give rise to a multitude of different cell types and organs which will acquire different functions by expressing different sets of genes [2]. These modules are known to play a key role in the regulation of the transcription process, for example in Human [3] and in Drosophila [4].

Here we summarize the main features of CRMs. First, they contain several short (6–15 bp) DNA motifs that act as binding sites for transcription factors (TFBSs) and often allow different nucleotides at some of the binding positions. In other words, there may be mutations on TFBSs. Second, these TFBSs act seemingly independently of the distance and orientation to their target genes as a consequence of looping. It follows that the strand to which a CRM under study belongs is unknown so both cases need to be considered. Third, they maintain their functions independently of the sequence context, are modular and contribute additively and partly redundantly to the overall expression pattern of their target genes. Finally, enhancers with similar transcription factors binding sites content have a high probability of bearing a similar function. This is why predictions and classifications of enhancers can be addressed by similarity searches. However, the presence of multiple binding sites, with different spacing between them, can make the comparison of two CRMs very difficult. For these reasons biologists need first to screen ChIP-seq datasets to select cell-specific regulatory sequences on the basis of common contents.

A similarity measure for regulatory sequences is crucial to detect and understand functional similarities between two enhancers and will facilitate large-scale analyses like clustering, prediction and classification. As opposed to traditional methods that output a list of putative TFBSs, alignment-free methods [5–7] do not try to find any candidates. Instead, they analyze many long regulatory regions, which are composed by several TFBSs along with the background, in order to group together those sharing a similar content in terms of TFBSs. If the identification and positioning of TFBSs are of concern, then well-known tools like MotifSampler [8] can be applied as a post-process.

The comparison of sequences can be carried out without the need of costly alignments. A sequence can be represented by its word distribution. It has been shown that the word content and distribution can be effectively used to compare sequences in a number of applications [9]. This recent research field is usually referred as alignment-free. In the context of CRMs, where it is assumed that a similar function is driven by the presence of different binding site contents, the idea to describe a sequence by its word distribution still works just as well. In addition, alignment-free methods are receiving increasing attention because they are computationally efficient and can provide attractive alternatives when alignment-based approaches fail. For example the study of organism evolution using whole-genome sequence is impossible to conduct with traditional alignment techniques [10, 11]. Similarly, the comparison of genomes from next-generation sequencing data can be performed only with alignment-free methods [12–14]. Several alignment-free methods have been devised for the identification of cis-regulatory modules [5–7].

In general alignment-free method are based on statistics of words with fixed-length *k*. The problem with these methods is that the performance depends dramatically on the choice of the resolution *k* [10]. For example in the analysis of enhancers using simulated data [5, 6], the best performing *k* is usually equal to the length of the implanted TFBS. In real cases its choice is critical because it is not possible to know the enhancer length in advance. Moreover, in the presence of several TFBSs, it is simply not feasible to select the *k* that best fits enhancers of different lengths. The statistical profile of variable length words in known CRMs has been used for the identification of potential CRMs in [15]. However, this method is supervised, in the sense that it uses orthologs of the known CRMs. In this paper we extend the idea of alignment-free measures accounting for multiple resolutions and without depending neither on any knowledge nor accurate prediction of TFBSs.

The Entropic Profile (EP) is a function of the genomic location that captures the importance of that region with respect to the whole genome [16, 17]. This method proved useful for the identification of conserved genomic regions. The score *EP* is based on the distribution of variable length words. For each position, it computes a function that represents the deviation from the known distribution. This function is a good candidate to be transformed into an alignment-free measure based on variable length word counts. However, *EP* can be computed only for a single sequence, and it cannot be directly applied as a mean for comparison. The main contributions of this paper are the followings:

- we extend the function *EP* for pairwise sequence comparison;
- as a byproduct, given that the word counts are not independent because of overlaps, we provide a formula for computing the exact variance of variable length word counts;
- we will show that pairwise sequence similarity of regulatory sequences is able to estimate similar in vivo activity.

In the next Sections “Previous work on alignment-free measures” and “Entropic profiles” we review the previous work on alignment-free statistics and present the original definition of Entropic Profile. Then, in Section “Methods”, their statistical properties are studied and particular attention is paid to the role of the variance. The extension of the well-known alignment-free measures is discussed in Section “New alignment-free measures derived from Entropic Profiles”, and implemented in a tool called EP_sim. In Section “Results and Discussion” the results are discussed and compared with the state of the art. Conclusions and future work are reported in Section “Conclusions”.

### Previous work on alignment-free measures

The common way to identify homologous sequences is sequence alignment, for which many algorithms have been proposed in literature [18, 19]. Nevertheless they are unsuitable for predicting and classifying enhancers through the matching of transcription factor binding sites for many reasons [9, 20]:

- transcription factor binding sites are short motifs so they frequently match to genomic or even random DNA sequences so enhancer similarity or dissimilarity may be due primarily to their background;
- enhancer location and orientation do not matter so no reliable alignment can be obtained;
- they are time-consuming and inadequate for comparing sequences in realistically large datasets, e.g. large ChIP-seq datasets;
- enhancers do not work alone and their coordinated action cannot be fully explored with a single alignment.

On the contrary, alignment-free approaches provide viable alternatives [9, 20]. With the aim of effectively summing up sequence content they are usually based on *k*-mer counts.

Historically, *D*_2_ [21], see Formula 1, is one of the first proposed similarities and is defined as the inner product of the *k*-mer frequency vectors. Consider two genome sequences *A* and *B*, of length *n*, and let *A*_*w*_ and *B*_*w*_ be the frequencies of word *w*, of length *k*, in *A* and *B*. Let $\tilde {A}_{w}=A_{w} - (n-k+1)*p_{w}$, where *p*_*w*_ is the probability of *w* under the null model. Despite its simplicity and distance properties, *D*_2_ can be dominated by the noise caused by the randomness of the background and has low statistical power to detect potential relationship. As a result, more powerful variants, ${D_{2}^{S}}$ and $D_{2}^{*}$ [22], see Formulas 2 and 3, have been developed by standardizing the *k*-mer counts with their expectations and standard deviations.

(1) $$  D_{2} = \sum_{w} A_{w} B_{w}  $$

(2) $$  {D_{2}^{s}} = \sum_{w \in \Sigma^{k}} \frac{\tilde{A}_{w} \tilde{B}_{w}}{\sqrt{\tilde{A}^{2}_{w} + \tilde{B}^{2}_{w}}}  $$

(3) $$  D_{2}^{*} = \sum_{w \in \Sigma^{k}} \frac{\tilde{A}_{w} \tilde{B}_{w}}{(n-k+1)p_{w}}.  $$

An implementation of *D*_2_, $D_{2}^{*}$ and ${D_{2}^{S}}$ is provided by ALF [5], which, by default, uses another similarity measure named *N*_2_, one of the best available methods for the analysis of regulatory sequences. *N*_2_ aims at overcoming the limitation of exact word counts by taking into account word neighbourhood counts. *N*_2_ is defined similarly to $D_{2}^{*}$ except that every word *w* is replaced with a set *n*(*w*) of words somehow linked to *w*, e.g. reverse complement and mismatches.

Several other alignment-free statistics have been proposed recently for different applications: multiple alignment [23], phylogeny [11, 24], classification of NGS data [12, 13], reads clustering [25, 26], and many others.

The major drawback of alignment-free measures is that they are all tied on the choice of the resolution *k*, which crucially influences performances but cannot be known in advance. Entropic Profiles, which are based on variable length word counts by definition, can be extended to create new alignment-free measures accounting for multiple resolutions. In particular we will show that Entropic Profiles pave the way to more robust but still efficient alignment-free methods.

### Entropic profiles

The concept of Entropic Profiler (EP) was introduced to analyze DNA sequences, in particular to detect exceptional motifs [16]. The Entropic Profiler takes a genome in input and evaluates a function of the genomic location that captures the importance of that region with respect to the whole genome. It proceeds through three steps. First, it calculates the distribution of each word up to a maximum length. Second, for each position in the genome, it evaluates a function based on the distribution of the words ending there with length up to the maximum. Third, for each position, it computes the z-value representing the deviation of that position from the known distribution. This score is based on the Shannon entropies of the word distribution. The formal definition of entropic profiles [16, 17] comes from the use of the CGR representation to estimate the sequence Renyi entropy on the basis of the Parzen window density estimation method. The *EP* is defined for every location *i* of the entire sequence *S* as:

(4) $$  \hat{f}_{L,\varphi}(x_{i})=\frac{1+\frac{1}{l}\sum_{k=1}^{L} 4^{k}\varphi^{k}\cdot c\left(\left[i-k+1, i\right]\right)}{\sum_{k=0}^{L} \varphi^{k}}  $$

where *l* is the length of the entire sequence, *L* the resolution, i.e. the *k*-mer length, *φ* is a smoothing parameter, and *c*([*i*−*k*+1,*i*]) is the number of occurrences of $x_{i-k+1}\dots x_{i}$, i.e. the suffix of length *k* that ends at position *i*.

*EP* values are standardized with their arithmetic mean *m*_*L*,*φ*_ and standard deviation *s*_*L*,*φ*_:

(5) $$  EP_{L,\varphi}(x_{i}) = \frac{\hat{f}_{L,\varphi}(x_{i})-m_{L,\varphi}}{s_{L,\varphi}} \text{, where}  $$

(6) $$  m_{L,\varphi}=\frac{1}{l}\sum_{i=1}^{l} \hat{f}_{L,\varphi}(x_{i})  $$

(7) $$  s_{L,\varphi}=\sqrt{\frac{1}{l-1}\sum_{i=1}^{l} \left(\hat{f}_{L,\varphi}(x_{i})-m_{L,\varphi}\right)^{2}}  $$

Entropic Profilers proved to be useful for the discovery of patterns in genome [17] and they can be computed efficiently in linear time and space [27–29]. By definition Entropic Profiles are based on multiple resolution *k*-mers counts, thus they are not tied to a fixed resolution *k*, as almost all alignment-free measures. Our intent is to extend this function for developing new alignment-free measures for the prediction and classification of enhancers.

## Methods

### From Entropic Profiles to multiple resolution alignment-free measures

In order to establish a suitable alignment-free measure, first we need to study the statistical properties of Entropic Profiles. We can simplify the original Formula 4 and consider the main term, that we call simple entropy *SE*_*w*_ of a word *w*=(*w*_1_,…,*w*_*L*_) of length *L* :

(8) $$ SE_{w} = \frac{\sum_{k = 1}^{L} a_{k} c_{w, k} }{\sum_{k = 1}^{L} a_{k}}  $$

where *c*_*w*,*k*_ is the number of occurrences of the *k*-mer suffix *s*_*w*,*k*_ and the weights *a*_*k*_ have been generalized.

The statistical properties of *SE*_*w*_ have not been carefully studied yet. In the previous works [27], only the expectation of this function has been explored. In addition, in [16, 17], the standardization is done with respect to the arithmetic mean and standard deviation (see Formula 6 and 7). This procedure can introduce biases due to the noise present in the input sequence. Indeed, the standardization does not depend on the word *w* that we want to score, but instead it is applied regardless of the particular word *w*, see Formula 5 where mean and variance are computed once and for all from the sequence under examination. Different words have different probability to occur, for example the string *AAAA* has more chance to appear than *ACGT*, because of its autocorrelation. Thus the number of occurrences of a word should be standardized with respect to the word statistics, as in $D_{2}^{*}$ already reported in Formula 3. In order to replicate the same scheme we first need to study the statistical properties of the simple entropy *SE*_*w*_.

### Computing the expected entropy

Without loss of generality the entire sequence $S = \left (X_{1}, X_{2},\ldots, X_{i},\ldots, X_{l}\right)$ can be modeled by a stationary Markov chain [30]. Here, we use a first-order Markov chain, but all results can be extended to any other order. Thanks to the stationarity of the Markov chain, the probability *μ*(*w*) that a word *w* occurs does not depend on the position *i*, and it is: $ \mu (w) = \mu \left (w_{1}\right) \prod _{j=2}^{L} \pi \left (w_{j-1}, w_{j}\right)$, where $\mu \left (w_{1}\right)$ is the probability that the first letter occurs and *π*(*w*_*j*−1_,*w*_*j*_) is the transition probability from letter *w*_*j*−1_ to *w*_*j*_.

It is useful to define the variable *Y*_*i*_(*w*), which indicates if *w* occurs at position *i*:

(9) $$  {}Y_{i}(w) = \left\{\begin{array}{ll} 1, &\quad \text{if (\(X_{i}\), \(X_{i+1},\ldots,X_{i+L-1}\)) \!= \!(\(w_{1}\), \(w_{2},\ldots,w_{L}\)),} \\ 0, &\quad \text{otherwise.} \end{array}\right.  $$

For each *i*, *Y*_*i*_(*w*) is a Bernoulli variable with parameter *μ*(*w*) so its expectation is *E*[*Y*_*i*_(*w*)]=*μ*(*w*) and its variance is *Var*[*Y*_*i*_(*w*)]=*μ*(*w*)[1−*μ*(*w*)]. This indicator provides a way to define the number of occurrences *c*_*w*_ of word *w*: $ c_{w} = \sum _{i = 1}^{l - L + 1} Y_{i}(w)$.

Now, based on the variables *Y*_*i*_(*w*), the expected entropy *E*[*SE*_*w*_] of the word *w* can be defined as in the following:

$$E[SE_{w}] = E\left[\frac{\sum_{k = 1}^{L} a_{k} c_{w, k} }{\sum_{k = 1}^{L} a_{k}}\right] = \frac{\sum_{k = 1}^{L} a_{k} E\left[c_{w, k}\right] }{\sum_{k = 1}^{L} a_{k}}$$

where

$$ E[c_{w, k}] = (l - k + 1) \mu(s_{w, k}) $$

Note that, as opposed to Formula 3, where the expected number of occurrences of the word *w* is estimated as (*l*−*k*+1)*μ*(*w*) (see definition of $\tilde {A}_{w}$), here *SE*_*w*_ accounts for multiple words of different lengths, and thus its expectation is computed accordingly.

### Computing the variance of entropy

In this section we continue to study the statistical property of entropies *SE*_*w*_. If we consider the standardization proposed in Formula 3, we can note that the denominator does not contain the exact variance but an approximation. The variance is replaced by the estimated mean of the word occurrence across the two sequences. If the probability of the word pattern is small, this approach can be justified by considering a Poisson approximation for the individual word counts. Here instead we are interested in deriving the exact variance of entropies *SE*_*w*_.

The variance *Var*[*SE*_*w*_] is important to take into account the dependence between entropies of overlapping words:

$$\begin{aligned} &Var[SE_{w}] = Var\left[ \frac{\sum_{k = 1}^{L} a_{k} c_{w, k} }{\sum_{k = 1}^{L} a_{k}} \right] \\ &\qquad \! \qquad= \frac{\sum_{k' = 1}^{L} \sum_{k^{\prime\prime} = 1}^{L} a_{k'} a_{k^{\prime\prime}} Cov\left[c_{w, k'}, c_{w, k^{\prime\prime}} \right]}{(\sum_{k = 1}^{L} a_{k})^{2}} \end{aligned}$$

where the derivation of the covariance of the counts is non-trivial. There are two cases which need to be explored. If $k' = k^{\prime \prime } \equiv k$ there is only one suffix of fixed length, and $Cov\left [c_{w, k'}, c_{w, k^{\prime \prime }}\right ] = Var[c_{w, k}]$. Otherwise, if $s_{w, k'} \neq s_{w, k^{\prime \prime }}$, one word is the suffix of the other. For the first case we need to extend and adapt the formula for *Var*[*c*_*w*_] in [30]. The latter case is more involving because it deals with overlapping words of variable lengths. Here below we provide the exact formulas of the two cases.

#### Case 1: variance of the count

If $k' = k^{\prime \prime } \equiv k$, the covariances can be simplified as $Cov\left [c_{w, k'}, c_{w, k^{\prime \prime }}\right ] = Var[c_{w, k}]$. From [30], in order to derive *Var*[*c*_*w*,*k*_] we need to sum three terms which respectively take into account:

1. self-overlap of the word with itself;
2. partial self-overlap, the suffix of the word with its prefix or vice-versa;
3. disjoint occurrences.

Formally:

$${} Var[\!c_{w, k}] \!= \!(l-k+1) \mu(w) (1\! - \mu(w)) + 2 \mu\!(w)\! \sum_{d = 1}^{k - 1} \!(l-k-d+1\!) * $$

$$*\left[ \varepsilon_{k - d}(w) \prod_{j=k-d+1}^{k} \pi(w[j-1], w[j]) - \mu(w) \right] $$

$$ + 2 \mu^{2}(w) \sum_{t = 1}^{l-2k+1} (l-2k-t+2) \left[ \frac{\pi^{t}(w[k], w[1])}{\mu(w[1])} - 1 \right] $$

where *ε*_*u*_(*w*) is the asymmetric overlap indicator

$$\varepsilon_{u}(w) = \left\{\begin{array}{ll} 1 & \qquad \text{if}\quad\text{w[k-u+1\ldots k] = w[1\ldots u]} \\ 0 & \qquad \text{otherwise} \end{array}\right. \text{,}$$

and *t*=*d*−*k*+1 and *π*^*t*^(*w*[*k*],*w*[1]) is the probability that the last letter of *w* is separated from an occurrence of *w*[1] by *t*−1 letters.

#### Case 2: covariance of the counts of words of different length

In this second case, $w' = s_{w, k'} \neq w^{\prime \prime } = s_{w, k^{\prime \prime }}$ so one word is the suffix of the other. First of all, it can be assumed that $|w^{\prime \prime }| = k^{\prime \prime } < |w'| = k'$ so, in this case, $w^{\prime \prime }$ is a suffix of *w*^′^. This assumption is without loss of generality because of the symmetry of the covariance, $Cov\left [c_{w, k'}, c_{w, k^{\prime \prime }}\right ] = Cov\left [c_{w, k^{\prime \prime }}, c_{w, k'}\right ]$. For simplicity of notation, let $c_{w, k'} = c_{w'}\phantom {\dot {i}\!}$ and $c_{w, k^{\prime \prime }} = c_{w^{\prime \prime }}$. The covariance can be expressed with respect to the random indicator variables, *Y*_*i*_(*w*), and developed by applying its well-known properties:

$$ Cov\left[c_{w, k'}, c_{w, k^{\prime\prime}}\right] = Cov\left[c_{w'}, c_{w^{\prime\prime}}\right] =$$

$${\footnotesize{} \begin{aligned} = Cov\left[\sum_{i = 1}^{l - k' + 1} Y_{i}(w'), \sum_{j = 1}^{l - k^{\prime\prime} + 1} Y_{j}(w^{\prime\prime})\right] = \sum_{i = 1}^{l - k' + 1} \sum_{j = 1}^{l - k^{\prime\prime} + 1} Cov\left[ Y_{i}(w'), Y_{j}(w^{\prime\prime})\right]= \end{aligned}}$$

(10) $$ {\footnotesize{} \begin{aligned}  =\sum_{i = 1}^{l - k^{\prime\prime} + 1} \sum_{j = 1, j \neq i}^{l - k^{\prime\prime} + 1} Cov\left[ Y_{i}(w'), Y_{j}(w^{\prime\prime})\right] + \sum_{h = 1}^{l - k^{\prime\prime} + 1} Cov\left[ Y_{h}(w') Y_{h}(w^{\prime\prime})\right] \end{aligned}}  $$

**Fig. 1:**
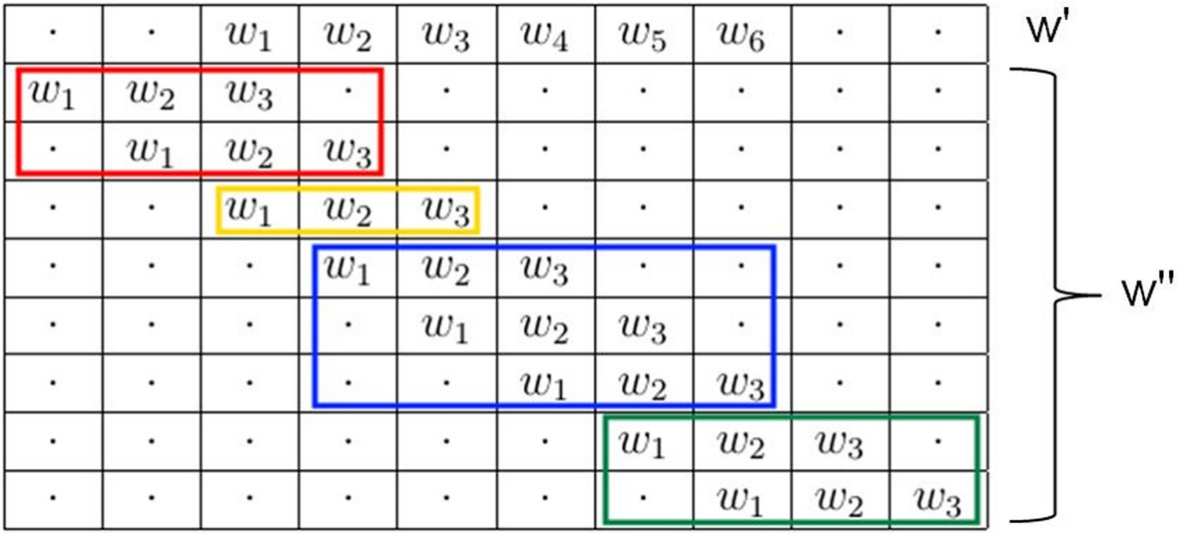
Possible overlaps between *w* ^′^ and $w^{\prime \prime }$

Note that the indices vary between 1 and $l - k^{\prime \prime } + 1$, so the last $k' - k^{\prime \prime }$ values of *Y*_*i*_(*w*^′^) are all zero since there are not enough letters to make the word *w*^′^. The two terms in Formula 10 can be interpreted as follows:

1. the former stands for all the terms due to two words of different length that do not start at the same position;
2. the latter stands for all the terms due to two words of different length that start at the same position (yellow words in Fig. 1).

To reformulate the former and to study overlaps, we can always fix the first *w*^′^ (the longest) and move $w^{\prime \prime }$ (the shortest, i.e. its suffix). In particular, let *d* be the shift of the moving word $w^{\prime \prime }$ with respect to the fixed word *w*^′^. A summary of the possible overlaps between *w*^′^ and $w^{\prime \prime }$ is shown in Fig. 1, so as to make the subsequent analysis of the two parts easier.

Part 1 of Eq. 10 can be reformulated by exchanging the sums over *i* and *d*. This way, *i* is fixed and *d* varied in order to consider the positions before *i* (left overlap) and after (right overlap).

$$\sum_{i = 1}^{l - k^{\prime\prime} + 1} \sum_{j = 1, j \neq i}^{l - k^{\prime\prime} + 1} Cov\left[ Y_{i}(w'), Y_{j}(w^{\prime\prime})\right] =$$

$${\footnotesize{} \begin{aligned} = \sum_{i = 1}^{l - k^{\prime\prime} + 1}\left(\sum_{d = 1}^{i-1} Cov\left[ Y_{i}(w'), Y_{i-d}(w^{\prime\prime})\right] + \sum_{d = 1}^{l-k^{\prime\prime}+1-i} Cov\left[ Y_{i}(w'), Y_{i+d}(w^{\prime\prime})\right]\right)= \end{aligned}}$$

$${\footnotesize{} \begin{aligned} = \sum_{d = 1}^{l - k^{\prime\prime}}\left(\sum_{i = d + 1}^{l-k^{\prime\prime}+1} Cov\left[ Y_{i}(w'), Y_{i-d}(w^{\prime\prime})\right] + \sum_{i = 1}^{l-k^{\prime\prime}+1-d} Cov\left[ Y_{i}(w'), Y_{i+d}(w^{\prime\prime})\right]\right) \end{aligned}}$$

The last formula has been rewritten to highlight the left and right overlaps. Note that the second part 2 of equation 10 simply represents the case *d*=0.

Under a first-order Markov model (or greater), the indicators *Y*_*i*_(*w*^′^) and $Y_{j}(w^{\prime \prime })$ are not independent, not even if the corresponding positions are more than *k*^′^ letters away from each other [30]. Thus,

$${} Cov\left[ \!Y_{i}(w'), \!Y_{j}(w^{\prime\prime})\right] \,=\, E \! \left[Y_{i}(w')\! Y_{j}(w^{\prime\prime})\right] - E\! \left[Y_{i}(w')\right]\!E\! \left[Y_{j}(w^{\prime\prime})\right] $$

may be different from zero. Especially, there are three cases (see again Fig. 1):

- left shift, *d*≥1 (red words);
- right shift, *d*≥1 (blues and green words);
- zero shift, *d*=0 (yellow word).

##### Left shift

This case is represented in red in Fig. 1.

$$ {{} \begin{aligned} Cov\left[ Y_{i}(w'), Y_{i-d}(w^{\prime\prime})\right] = E\left[Y_{i}(w') Y_{i-d}(w^{\prime\prime})\right] - E\left[Y_{i}(w')\right]E\left[Y_{i-d}(w^{\prime\prime})\right] \end{aligned}}$$

where the first term comprehends two parts that respectively represent:

1. prefix - suffix overlap: two overlapping words, the latter of which (red words in Fig. 1) starts before the beginning and ends before the end of the former.
2. two non overlapping words.

Thus we can write:

$${{\kern-16.5pt}\begin{aligned} E[\!Y_{i}(w') Y_{i-d}(w^{\prime\prime})] \,=\,\! \left\{\!\!\begin{array}{ll} \varepsilon_{k^{\prime\prime}-d}^{left}(w^{\prime\prime}\!, \!w')\, \mu(w^{\prime\prime})\! \prod_{j=k'-k^{\prime\prime}\!-d+1}^{k'} \!\pi\!(w'_{j-1},\!w'_{j}) & \! \text{if}\, 1 \!\leq\! d \!<\! k^{\prime\prime} \\ \mu(w^{\prime\prime}) \mu(w') \left[\frac{\pi^{d-k^{\prime\prime}+1}(w^{\prime\prime}_{k^{\prime\prime}}, w'_{1})}{\mu(w'_{1})}\right] &\! \text{if}\, d \geq k^{\prime\prime} \\ \end{array}\right.\end{aligned}} $$

where $\varepsilon _{u}^{left}(w^{\prime \prime }, w')$ is the asymmetric overlap indicator

(11) $${} \varepsilon_{u}^{left}(w^{\prime\prime}, w') = \left\{\begin{array}{ll} 1 & \text{if}\, w^{\prime\prime}[k^{\prime\prime}-u+1\ldots k^{\prime\prime}] = w'[1\ldots u] \\ 0 & \text{otherwise} \end{array}\right.  $$

Since the expectation does not depend on the position *i* we can write:$E[Y_{i}(w')]E[Y_{i-d}(w^{\prime \prime })] = \mu (w')\mu (w^{\prime \prime })$.

##### Right shift

Analogously (but not symmetrically),

$${{} \begin{aligned}Cov\left[ Y_{i}(w'), Y_{i+d}(w^{\prime\prime})\right] = E\left[Y_{i}(w') Y_{i+d}(w^{\prime\prime})\right] - E\left[Y_{i}(w')]E[Y_{i+d}(w^{\prime\prime})\right]\end{aligned}}$$

where the first term comprehends three parts that respectively represent:

1. substring - string overlap: two overlapping words, the latter (blue words in Fig. 1) starts after the beginning and ends before the end of the former.
2. substring - prefix overlap: two overlapping words, the latter (green words in Fig. 1) starts before the end of the former and ends after it.
3. two non overlapping words.

$${\footnotesize{}</p><p class="noindent">\begin{aligned} E[\!Y_{i}(w')\! Y_{i+d}\!(w^{\prime\prime})\!] \,=\, \left\{\!\!\begin{array}{ll} \varepsilon_{k'-d}^{right}(w', w^{\prime\prime}) \mu(w') & \!\text{if}\, 1\!\leq\! d\! \leq\! k'\! \,-\, k^{\prime\prime} \\ \mu\!(w') \varepsilon_{k'-d}^{right}\!(w', w^{\prime\prime})\! \prod_{j=k'\!-d+1}^{k^{\prime\prime}}\! \pi\! \!\left(\!w^{\prime\prime}_{j-1},\!w^{\prime\prime}_{j}\!\right) &\!\text{if}\, k' \!\!- \!k^{\prime\prime} \!\!<\! d \!< \!k' \\ \mu(w') \mu(w^{\prime\prime}) \left[\frac{\pi^{d-k'+1}(w'_{k'}, w^{\prime\prime}_{1})}{\mu(w^{\prime\prime}_{1})}\right] & \text{\!if}\, d \geq k' \\ \end{array}\right. \end{aligned}}$$

where $\varepsilon _{u}^{right}(w', w^{\prime \prime })$ is the asymmetric overlap indicator

(12) $$ {{} \begin{aligned} \varepsilon_{u}^{right}(w', w^{\prime\prime}) = \left\{\begin{array}{ll} 1 &\quad \text{if}\, u < k^{\prime\prime} \land w'[k'-u+1\ldots k'] = w^{\prime\prime}[1\ldots u] \\ 1 &\quad \text{if}\, u \geq k^{\prime\prime} \land w^{\prime\prime}\, \text{is a substring of}\, w' \\ 0 &\quad \text{otherwise} \end{array}\right. \end{aligned}}  $$

##### Zero shift

This case considers the prefix - string overlap, in other words two overlapping words starting at the same position the latter of which ends before the end of the former.

$${\footnotesize{} \begin{aligned} E\!\left[Y_{h}(w') Y_{h+d}(w^{\prime\prime})\right] \!= \!E\left[Y_{h}(w') Y_{h+0}(w^{\prime\prime})\right] \,=\, \mu(w')\!*\!1\!\! +\! (1\,-\,\mu(w'))*0\! =\! \mu(w') \end{aligned}}$$

Finally, we can put them all together so as to derive the exact formula for the covariance of the counts of two words with different length:

$${} Cov\left[c_{w, k'}, c_{w, k^{\prime\prime}}\right] = (l - k^{\prime\prime} + 1)(\mu(w') - \mu(w')\mu(w^{\prime\prime})) + $$

$${} + \sum_{d = 1}^{k'-k^{\prime\prime}} (l - k^{\prime\prime} + 1 - d) \mu(w') \left(\varepsilon_{k'-d}^{right}(w', w^{\prime\prime}) - \mu(w^{\prime\prime})\right) + $$

$${\footnotesize{} \begin{aligned} +\! \sum_{d = k'-k^{\prime\prime}+1}^{k'} \!(l \,-\, k^{\prime\prime} \!+ \!1 \,-\, d) \mu(w')\! \left[ \!\varepsilon_{k'-d}^{right}(w', w^{\prime\prime})\! \prod_{j=k'-d+1}^{k^{\prime\prime}} \!\pi\! \!\left(w^{\prime\prime}_{j-1},w^{\prime\prime}_{j}\right) \,-\, \mu(w^{\prime\prime})\!\right] \!+ \end{aligned}} $$

$${\footnotesize{} \begin{aligned} + \sum_{d = 1}^{k^{\prime\prime}} (l-k^{\prime\prime}+1\!-d) \mu(w^{\prime\prime})\! \left[ \!\varepsilon_{k^{\prime\prime}-d}^{left}(w^{\prime\prime}, w')\! \prod_{j=k'-k^{\prime\prime}-d+1}^{k'} \!\pi \!\!\left(w'_{j-1},w'_{j}\right) \,-\, \mu(w')\!\right] \!+ \end{aligned}} $$

$${} +\! \sum_{d = k^{\prime\prime}}^{l-k^{\prime\prime}} \!(l-k^{\prime\prime}+1-d) \mu(w^{\prime\prime}) \mu(w')\! \left[\frac{\pi^{d-k^{\prime\prime}+1}\left(w^{\prime\prime}_{k^{\prime\prime}}, w'_{1}\right)}{\mu\left(w'_{1}\right)} - 1\!\right] \!+ $$

$${} + \sum_{d = k'}^{l-k^{\prime\prime}} (l-k^{\prime\prime}+1-d) \mu(w') \mu(w^{\prime\prime}) \left[\frac{\pi^{d-k'+1}\left(w'_{k'}, w^{\prime\prime}_{1}\right)}{\mu\left(w^{\prime\prime}_{1}\right)} - 1\right] $$

This is the exact formula that, together with the other case, can be used to compute the variance of *SE*_*w*_. Unlike previous approaches that approximate the variance of equal length word counts, we have also provided a challenging formula for computing the exact variance of variable length word counts. For the sake of simplicity, as done in [5], the last two terms, i.e. the non-overlapping terms, will be neglected thereby assuming that the occurrence of non-overlapping words is independent of the sequence in between.

We believe that this result can be of general interest, and that it can be used also in other applications. For example exact word statistics are fundamental for the discovery of surprising/over-represented patterns [30, 31].

### New alignment-free measures derived from Entropic Profiles

Entropies and counts are very much alike, as already described in the previous section. The basic intuition is that Entropic Profiles can be used instead of *k*-mer counts, so that one can build alignment-free statistics that are not based on the fixed length *k*, but that are multiple resolution. This suggests that the adaptation of the state-of-the-art measures can be done by replacing the vector of *k*-mer counts with the vector of entropies.

Consider two genome sequences *A* and *B* and let $A_{SE_{w}}$ and $B_{SE_{w}}$ be the entropies of word *w* in *A* and *B*. We can redefine classical alignment-free measures as:

(13) $$  EP_{2} = \sum_{w} A_{SE_{w}} B_{SE_{w}}  $$

(14) $$  EP_{2}^{*} = \sum_{w} \frac{\left(A_{SE_{w}} - E\left[A_{SE_{w}}\right]\right) \left(B_{SE_{w}} - E\left[B_{SE_{w}}\right]\right)}{{Var\left[AB_{SE_{w}}\right]}}  $$

While the implementation of *EP*_2_ is straightforward, $EP_{2}^{*}$ instead is based on the statistical properties of entropies. The theory developed in the previous section is preliminary to the implementation of $EP_{2}^{*}$.

Note that Entropic Profiles, expectations and variances can be pre-computed in linear time and space by adapting the implementation in [27]. Thus, the proposed statistics, as many others, can be computed efficiently.

We implemented these alignment-free measures, as well as traditional ones, in a software called *EP*-*sim* that is freely available^1^. It is based on the library SeqAn [32] that provides efficient string primitives. Among the different options available, the possibilities to include reverse complements and to compute an approximated version of the variance are of note. In particular one can extend the formulas for the mean and variance to include also reverse complements. There are several ways to incorporate reverse complements into the score. The method we selected consists in taking the maximum between the entropies of a word and its reverse complement. In practice the fact that only the strongest signal is taken makes the effect of exceptional words more incisive. This solution is only one of the possibilities. In *N*_2_ [5], the *k*-mer counts from the reverse and forward strand can be combined in many ways. There are four options: both-strands, to calculate the pairwise score using both strands from the input sequences, mean, min and max. In general, the use of reverse complements will be of help for the detection of enhancers and in other applications.

## Results and Discussion

This section deals with the testing procedures for the study of the statistical power of the proposed multi-resolution sequence similarity measures. The task of pairwise comparison of regulatory sequences is much harder than traditional pairwise alignment since only very few shared words might lead to a similar activity. In this section we want to test if pairwise sequence similarity of regulatory sequences is able to estimate similar in vivo activity.

The same biological problem has been addressed in [5–7] and we chose to compare with these methods using the same experimental setup. Here, we report experiments on simulated and real regulatory sequences, by using the same evaluation procedure. In each experiment two equal-length sets of sequences, which are named negative and positive set, are built. Sequences in the former are dissimilar while those in the latter similar. The positive predictive value (PPV) is evaluated in two steps: first similarity scores are computed for each pair of sequences in the two sets; then similarity scores are sorted in descending order, and the PPV is the percentage of pair of sequences from the positive set in the first half of the chart. The best PPV is 1 and means a perfect separation between negative and positive sets while a PPV close to 0.5 implies no statistical power. Performances will depend on the choice of the background model, the *k*-mer length and the weights *a*_*k*_. For the latter we will use a Gaussian kernel with standard deviation *σ*, which is centered about *k*=*L*, i.e. $a_{k} = e^{-\frac {(L - k)^ 2}{2 \sigma ^ 2}}$.

**Fig. 2:**
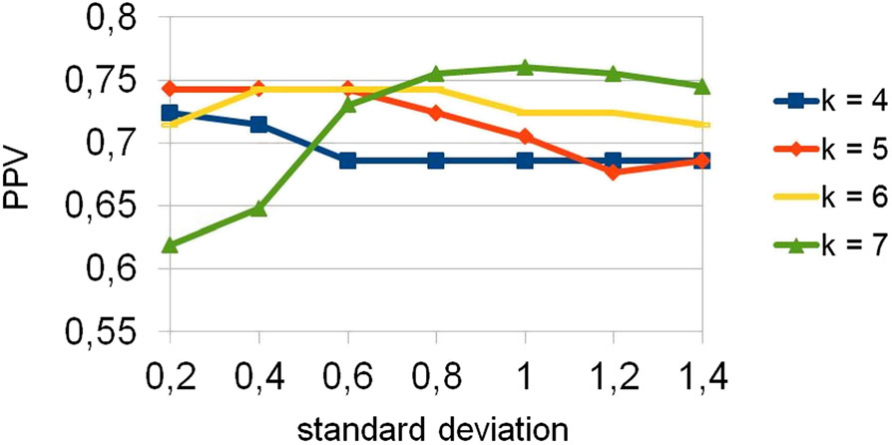
PPV as a function of standard deviation and *k*-mer length. In this experiment the sequence length is 500 and the insertion probability is 0.01. The implanted motifs are *AGCCA*, *G* *C* *C* *A*,*T* *A* *G* *C* *C* *A*,*C* *C* *A* *G*,*A* *G* *C* *C* *A* *G*

In order to study the influence of the parameter *σ* on the performance curves, we devise a simple test. First, we randomly generate a set of sequences as negative set, then we create the positive set by implanting a set of similar motifs, of average length 5 (*AGCCA*, *GCCA*, *TAGCCA*, *CCAG*, *AGCCAG*), in those of the negative set. Figure 2 shows the results of the study of the influence of the standard deviation. In this example the sequence length is 500 and the insertion probability 0.01. An high standard deviation positively impacts performances when the *k*-mer length is overestimated, because high values of the standard deviation make short motifs to have bigger weights. To exemplify the idea, if the standard deviation is 1.5, the four biggest weights are 1, 0.80, 0.41 and 0.13 and performances are influenced while if the standard deviation is 0.1, the Gaussian bell is so thin that $EP^{*}_{2}$ is equivalent to $D^{*}_{2}$. As expected the performances worsen when the *k*-mer length is underestimated.

### Implanted motifs on Drosophila genome

In this simulation study, the sequences in the negative set are randomly picked from a real genome while those in the positive set are built by implanting a set of motifs in those of the negative set, since random sequences are unrealistic backgrounds. Thus, as in [33], we chose the *Drosophila* genome, whose intergenic sequences, which are regions containing functionally important elements such as promoters and enhancers, are downloadable from FlyBase^2^. Patterns can be artificially implanted via the pattern transfer model [22] or the revised one [33] with the aim of mimicking the exchange of genetic material. While, under the former model, only strings of the same length, e.g 5, are considered, under the latter, also strings of different length, e.g. 4, 5 and 6 are implanted.

**Fig. 3:**
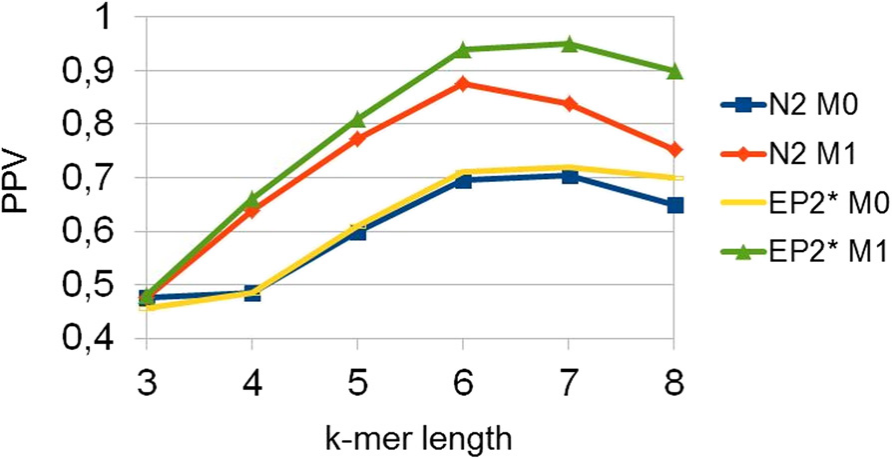
Background model M1 outperforms M0. In this experiment the sequences length is 2000, the standard deviation is 0.5, the insertion probability is 0.004 and the implanted motif is *AGCCAG*

The goal of this experiment is to assess the influence of the background model so as to use the best one in the next tests. It has been performed varying many parameters such as implanted motifs, insertion probability, entire sequence length and *k*-mer length. Generally, first-order Markov model (M1) outperforms the Bernoulli model (M0). This is outlined by Fig. 3, which shows performances as a function of background model and *k*-mer length. In this example, only one motif *AGCCAG*, of length 6, has been implanted, the insertion probability has been set to 0.004, the sequences length is 2000 and the standard deviation is 0.5. It is important to observe that $EP^{*}_{2}$ is better than *N*_2_ if the *k*-mer length is overestimated, i.e. *k*≥6, as a consequence of the multi-resolution property of entropic profiles.

**Fig. 4:**
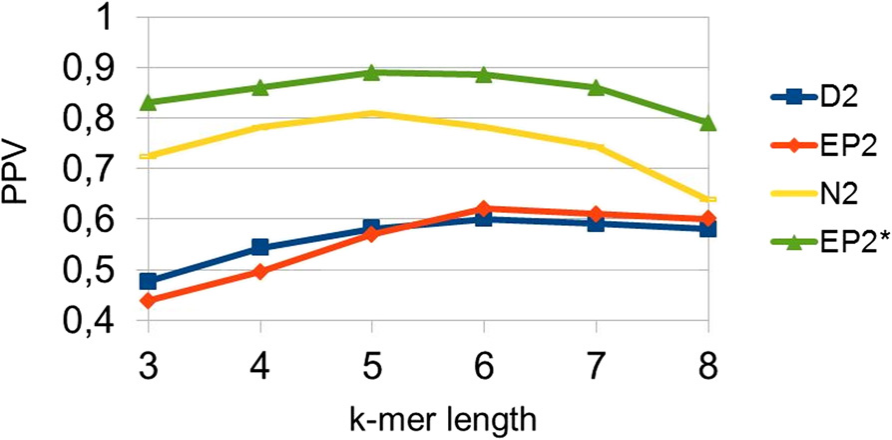
PPV as a function of *k*-mer length and method. In this experiment the sequence length is 4000, the standard deviation is 0.6, the insertion probability is 0.008 and the implanted motifs are *AGCCA*, *GCCA*, *TAGCCA*, *CCAG*, *AGCCAG*

**Fig. 5:**
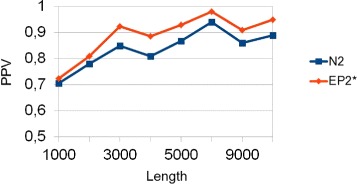
PPV as a function of entire sequence length and method. In this experiment *k*=5, the sequence length is 4000, the standard deviation is 0.6, the insertion probability is 0.008 and the implanted motifs are *AGCCA*, *GCCA*, *TAGCCA*, *CCAG*, *AGCCAG*

Considering our limited knowledge of regulatory sequences [5], it is interesting to evaluate performances when implanting similar motifs of different length via the more realistic pattern transfer model revised. The motifs implanted are similar to each other, in the sense that they share common subsequences (*AGCCA*, *GCCA*, *TAGCCA*, *CCAG*, *AGCCAG*), with average length of 5. We have performed many experiments varying both *k*-mer and entire sequence length. Figure 4 shows the results when the entire sequence length is 4000, the insertion probability 0.008 and the standard deviation 0.6. $EP^{*}_{2}$ outperforms *N*_2_ and both variants of *D*_2_, which do not take into account the statistical properties of counts or entropies, have no statistical power. The worse performance of *D*_2_ and *EP*_2_ are consistent throughout all experiments, thus we will concentrate on the comparison of $EP^{*}_{2}$ and *N*_2_. If a different set of motifs is implanted, the absolute performance can vary. However, the relative performance between the methods remains unaltered. In the previous Figure the pick is at *k*-mer length 5, which is the selected value for the next experiment. Figure 5 shows that these results hold also varying the entire sequence length. Performances tend to increase with the length of the sequence, because the number of implanted motifs also increases, as expected.

### Comparison of mouse regulatory sequences

The above simulations deal with artificial CRMs from unrelated sequences. The next series of experiments involves neither artificial enhancers nor implanted transcription factor binding sites. The positive set is build from ChIP-seq data of real enhancers, which have been already identified in a genome-wide manner using the co-activator protein p300 by [34, 35]. More precisely, it consists in sequences of length between 350 and 1000 that are issue-specific enhancers of mouse embryos active in one of the following tissues: forebrain, midbrain, limb or heart. These studies [34, 35] have identified 2543, 561, 2105 and 3597 peaks from forebrain, midbrain, limb and heart respectively. For the purpose of this study we select the top 200 peaks for each tissue.

**Fig. 6:**
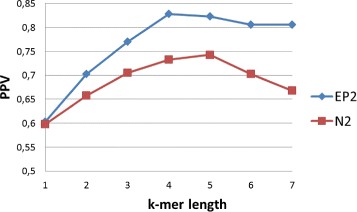
Comparison of mouse tissue-specific enhancers versus random mouse genomic sequences. Values in the graph represents the average PPV, for all tissues, for various *k*-mer lengths. In this experiment the standard deviation is 0.7

In the first experiment, we want to assess if in-vivo identified enhancers can be distinguished from random mouse genome sequences. To this end, the negative set contains sequences taken at random from the mouse genome, which is downloadable from Ensembl ^3^. To obtain accurate estimations, we calculated the average over 10 samples, each time drawing 20 sequences from the positive set of tissue specific enhancers. Using the same evaluation measures as in the previous section, we tested the ability of alignment-free sequence comparison methods to detect functional similarity of regulatory sequences. Given that no artificial motif is implanted, which implies that the best motif length is unknown and function of the tissue, the chosen standard deviation is 0.7 so short motifs have bigger weights. The purpose is to take advantage of the multi-resolution property. The results for $EP^{*}_{2}$ and *N*_2_, while varying the *k*-mer length, are reported in Table 1. A summary of the average over all tissues is in Fig. 6. In general the performance of $EP^{*}_{2}$ is better than *N*_2_ for different *k*-mer lengths. If one considers the statistics of single bases, *k*=1, regulatory sequences can be detected with a PPV of 60 *%*. Probably because the *GC* content of regulatory sequences is different from random mouse regions. If larger *k* are considered the performance of both methods increase up to a maximum obtained for *k*=4. It is interesting to note that, as the parameter *k* increases the performance of both methods worsen, however, due to the multi resolution property the PPV of $EP^{*}_{2}$ decreases less rapidly.

**Table 1:** Comparison of mouse tissue-specific enhancers versus random mouse genomic sequences. Values in the table represents the average PPV, over all tissues, varying the *k*-mer length. The standard deviation is 0.7

$EP^{*}_{2}$	***k*** **-mer length**						
**Tissue**	1	2	3	4	5	6	7
Limb	0.61	0.68	0.77	0.82	0.82	0.81	0.8
Forebrain	0.59	0.71	0.78	0.8	0.83	0.82	0.82
Midbrain	0.58	0.69	0.72	0.84	0.81	0.78	0.79
Heart	0.63	0.73	0.81	0.85	0.83	0.81	0.81
**Average**	**0.60**	**0.70**	**0.77**	**0.83**	**0.82**	**0.80**	**0.80**
***N***_***2***_	***k*** **-mer length**						
**Tissue**	1	2	3	4	5	6	7
Limb	0.6	0.66	0.71	0.74	0.75	0.69	0.66
Forebrain	0.59	0.68	0.7	0.73	0.76	0.72	0.68
Midbrain	0.58	0.63	0.68	0.71	0.72	0.69	0.65
Heart	0.62	0.66	0.73	0.75	0.74	0.71	0.68
**Average**	**0.6**	**0.66**	**0.70**	**0.73**	**0.74**	**0.70**	**0.67**

**Fig. 7:**
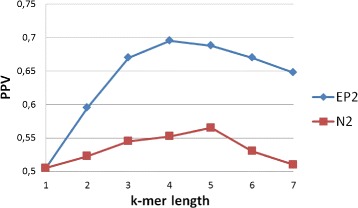
Comparison of mouse tissue-specific enhancers versus versus others tissue-specific enhancers. Values in the graph represents the average PPV, for all tissues, for various *k*-mer lengths. In this experiment the standard deviation is 0.7

The previous test shows that tissue-specific enhancers have similar word content. However, the comparison with random genomic sequences can be biased by the technology, e.g. when it more likely extracts sequences with high or similar GC-content, as already described in [33] and [5]. To avoid this bias, different regulatory sequences are compared with each other. In other words, the positive set contains the enhancers active in one of the tissues while the negative set contains the enhancers active in all the other. This is a much more challenging test, that can be used by biologists to select enhancers that drive a similar expression pattern. The results are averaged over 10 runs, the number of sequences per set is 35 and the standard deviation is 0.7 as before. The results in Table 2 show that $EP^{*}_{2}$ is again better than *N*_2_ for different *k*-mer lengths. However, in these experiments the frequency of single bases is not discriminative, unlike the previous tests. A comprehensive summary, for different *k*-mer length, can be found in Fig. 7. These plots show the performance of pairwise comparison with alignment-free methods for enhancers active in the same tissue versus enhancers active in different tissues. The performance is reduced compared to randomly selected genomic sequences. Nevertheless, enhancers active in the same tissue have higher pairwise scores.

**Table 2:** Comparison of mouse tissue-specific enhancers versus others tissue-specific enhancers. Values in the table represent the average PPV, over all tissues, varying the *k*-mer length. The standard deviation is 0.7

${EP^{*}_{2}}$	*k*-mer length						
Tissue	1	2	3	4	5	6	7
Limb	0.52	0.59	0.68	0.71	0.7	0.69	0.67
Forebrain	0.5	0.58	0.62	0.65	0.63	0.63	0.59
Midbrain	0.51	0.61	0.68	0.69	0.7	0.68	0.66
Heart	0.49	0.6	0.7	0.73	0.72	0.68	0.67
**Average**	**0.50**	**0.59**	**0.67**	**0.69**	**0.69**	**0.67**	**0.65**
*N*_2_	*k*-mer length						
Tissue	1	2	3	4	5	6	7
Limb	0.51	0.55	0.58	0.59	0.61	0.54	0.53
Forebrain	0.51	0.52	0.54	0.56	0.57	0.51	0.52
Midbrain	0.51	0.5	0.51	0.48	0.52	0.54	0.5
Heart	0.49	0.52	0.55	0.58	0.56	0.53	0.49
**Average**	**0.50**	**0.52**	**0.54**	**0.55**	**0.56**	**0.53**	**0.51**

These regulatory sequences can be further compared pairwise. Following the same setup as above, the pairwise comparison of all tissue-specific enhancers are shown in Table 3. Although the average results are similar to those of Table 2, the pairwise accuracy can vary greatly. Enhancers obtained from Forebrain and Midbrain tissues are difficult to be distinguished from other tissues. Interestingly Heart enhancers show greater similarities then all other enhancers. As reported in [35], the vast majority (84 %) of peaks in the heart enhancers do not overlap any of the other three tissues. These experiments confirm that similar tissue-specific enhancers have a higher sequence similarity, and thus they can be detected with alignment-free methods.

**Table 3:** Comparison of mouse tissue-specific enhancers with each other. Values in the table represent the average PPV, with *k*-mer length of 4 and standard deviation of 0.7

${EP^{*}_{2}}$	Limb	Forebrain	Midbrain	Heart
Limb	X	0.63	0.68	0.78
Forebrain	0.63	X	0.61	0.68
Midbrain	0.68	0.61	X	0.73
Heart	0.78	0.68	0.73	X
**Average**	**0.70**	**0.64**	**0.67**	**0.73**
*N*_2_	Limb	Forebrain	Midbrain	Heart
Limb	X	0.55	0.54	0.66
Forebrain	0.55	X	0.54	0.6
Midbrain	0.54	0.54	X	0.53
Heart	0.66	0.6	0.53	X
**Average**	**0.58**	**0.56**	**0.54**	**0.59**

### Speed tests

**Fig. 8:**
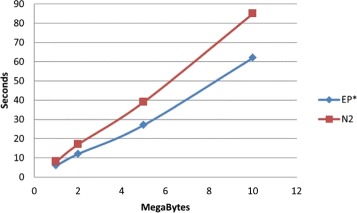
Running time as a function of the sequence length (k=7)

In this section we assess the performance, in terms of running time, of the two measures $EP_{2}^{*}$ and *N*_2_. For a given word *w*, both methods need to count not only the occurrences of *w*, but *N*_2_ considers also all words at Hamming distance 1 from *w*, whereas $EP_{2}^{*}$ sum up all suffixes of *w*. In the following experiments both methods include reverse complements as part of the occurrence counts. We create a dataset composed by 20 sequences taken at random from the mouse genome. All sequences have the same length and we test the running time while increasing the sequence length. The platform used for these experiments is a common laptop with Intel i7 and 4 GB of RAM. The results are summarized in Fig. 8. As expected the running time of both measures increases linearly with the length of the sequences. However, $EP_{2}^{*}$ is about 35 % faster than *N*_2_. This advantage is due to the fact that suffix counts can be easily recovered by exploiting word hashing properties.

## Conclusions

In this paper we studied the use of alignment-free measures to detect functional or evolutionary similarities among regulatory sequences. We introduced a multiple resolution alignment-free method based on Entropic Profiles that is designed around the use of variable-length words combined with statistical properties. To evaluate the performance of several alignment-free methods, we devised a series of tests on both synthetic and real data. In almost all simulations our method $EP^{*}_{2}$ outperforms all other statistics. Importantly $EP^{*}_{2}$ is also able to detect similarities between in vivo identified enhancer sequences, e.g. of mouse. This will help to better understand the sequence-dependent code within CRMs, which is responsible for the large diversity of cell types.

As a byproduct we provide a formula to compute the exact variance of variable length word counts, a result that can be of general interest also in other applications, e.g. the discovery of surprising patterns. As a future direction we plan to implement different methods to incorporate reverse complements. Another context where the these statistics can be of help is the comparison of viral sequences.

## Endnotes

^1^http://www.dei.unipd.it/~ciompin/main/EP-sim.html

^2^ FlyBase, http://flybase.org/

^3^ftp://ftp.ensembl.org/pub/release-84/variation/VEP/mus_musculus_vep_84_GRCm38.tar.gz
